# Understanding Family-Level Effects of Adult Chronic Disease Management Programs: Perceived Influences of Behavior Change on Adolescent Family Members' Health Behaviors Among Low-Income African Americans With Uncontrolled Hypertensions

**DOI:** 10.3389/fped.2018.00386

**Published:** 2019-01-10

**Authors:** Rachel L. J. Thornton, Tracy J. Yang, Patti L. Ephraim, L. Ebony Boulware, Lisa A. Cooper

**Affiliations:** ^1^Division of General Pediatrics and Adolescent Medicine, Department of Pediatrics, Johns Hopkins School of Medicine, Baltimore, MD, United States; ^2^Johns Hopkins Center for Health Equity, Baltimore, MD, United States; ^3^Department of Health, Behavior and Society at the Johns Hopkins Bloomberg School of Public Health, Baltimore, MD, United States; ^4^Welch Center for Prevention, Epidemiology and Clinical Research, Baltimore, MD, United States; ^5^New York-Presbyterian Morgan Stanley Children's Hospital, Columbia University Medical Center, New York, NY, United States; ^6^Division of General Internal Medicine, Department of Medicine, Johns Hopkins School of Medicine, Baltimore, MD, United States; ^7^Division of General Internal Medicine, Department of Medicine, Duke University School of Medicine, Durham, NC, United States

**Keywords:** cardiovascular diseases, family health, hypertension, health promotion, disease management, health disparities

## Abstract

**Background:** Despite improvements in cardiovascular disease (CVD) prevention and treatment, low-income African Americans experience disparities in CVD-related morbidity and mortality. Childhood obesity disparities and poor diet and physical activity behaviors contribute to CVD disparities throughout the life course. Given the potential for intergenerational transmission of CVD risk, it is important to determine whether adult disease management interventions could be modified to achieve family-level benefits and improve primary prevention among high-risk youth.

**Objective:** To explore mechanisms by which African-American adults' (referred to as index patients) participation in a hypertension disease management trial influences adolescent family members' (referred to as adolescents) lifestyle behaviors.

**Design/Methods:** The study recruited index patients from the Achieving blood pressure Control Together (ACT) study who reported living with an adolescent ages 12–17 years old. Index patients and adolescents were recruited for in-depth interviews and were asked about any family-level changes to diet and physical activity behaviors during or after participation in the ACT study. If family-level changes were described, index patients and adolescents were asked whether role modeling, changes in the home food environment, meal preparation, and family functioning contributed to these changes. These mechanisms were hypothesize to be important based on existing research suggesting that parental involvement in childhood obesity interventions influences child and adolescent weight status. Thematic content analysis of transcribed interviews identified both a priori and emergent themes.

**Results:** Eleven index patients and their adolescents participated in in-depth interviews. Index patients and adolescents both described changes to the home food environment and meal preparation. Role modeling was salient to index patients, particularly regarding healthy eating behaviors. Changes in family functioning due to study participation were not endorsed by index patients or adolescents. Emergent themes included adolescent care-taking of index patients and varying perceptions by index patients of their influence on adolescents' health behaviors.

**Conclusions:** Our findings suggest that disease management interventions directed at high-risk adult populations may influence adolescent family members' health behaviors. We find support for the hypotheses that role modeling and changes to the home food environment are mechanisms by which family-level health behavior change occurs. Adolescents' roles as caretakers for index patients emerged as another potential mechanism. Future research should explore these mechanisms and ways to leverage disease management to support both adult and adolescent health behavior change.

## Introduction

Preventing and treating child and adolescent obesity is essential for reducing cardiovascular disease (CVD) risk over the life course. An estimated 17% of U.S. children and adolescents are obese (1–3). Despite recent improvements in available CVD prevention and treatment (4), low-income African Americans experience excess CVD-related morbidity and mortality (4, 5). Excess obesity risk among low-income African-American children and adolescents contributes to disparities in lifetime CVD risk (6, 7). Furthermore, racial/ethnic and socioeconomic disparities in childhood obesity (8, 9) have not improved over the past decade (10), and may even be worsening (11, 12).

Lifestyle modifications aimed at weight loss and promoting healthy diet and physical activity (PA) behaviors are important for primary and secondary prevention of CVD and related morbidity in adults (13, 14) and adolescents (15). Parent and family involvement is important for preventing child and adolescent obesity and decreasing CVD risk over the life course. Parent and family involvement may also improve disease management outcomes among adults (7, 13, 16, 17). Yet, the potential for adult disease management interventions to achieve intergenerational benefits via family-level effects has not been extensively studied and may represent a missed opportunity to address CVD-outcome disparities and optimize primordial and primary prevention among high-risk youth.

Our overall goal was to investigate mechanisms by which adult disease management interventions may positively influence adolescent health behaviors. We did this by exploring the extent to which adults with uncontrolled hypertension (index patients) and their adolescent family members (adolescents) perceive *spillover* effects of participation in a blood pressure control disease management comparative effectiveness trial on adolescents' diet and physical activity behaviors. *Spillover* is a well-developed concept in family psychology, describing the notion that participation in one domain (e.g., self-care) can affect participation in another domain (e.g., family) (18–20).

For example, a parent's role as a participant or index patient in a disease management intervention could produce *spillover* to the family domain and elicit behavior change among other family members, including children or adolescents. Qualitative research suggests that dietary behavior change among adults with diabetes can produce changes in children's diet (21) and sugar sweetened beverage intake (22). Other studies have found that weight loss and behavior change interventions targeting overweight parents can simultaneously produce improvements in diet and physical activity behaviors for their children (23–25).

Adolescence is a critical window for intervention, with elevated BMI in adolescence associated with increased risk of premature death and CVD in adulthood (26). Increases in BMI during transition from adolescence to adulthood have accelerated in recent decades, particularly for African-American females (6). Research suggests interventions to improve healthy diets among adolescents should target parental diet and the home food environment (27, 28). Given the important relationship of the home environment with adolescent obesity and CVD risk and the role of adult family members as agents of change (29), CVD risk reduction interventions solely targeting adults may represent missed opportunities to simultaneously address adolescent CVD and obesity risk.

This work highlights the importance of developing strategies for modifying adult-focused disease management interventions to achieve positive family-level effects (30). Yet, a review by Barr-Anderson and colleagues (31) examining family-focused PA, diet, and obesity interventions in African-American girls found that, despite face validity to family engagement, there is a lack of clear evidence regarding how family engagement can best be used to achieve significant decreases in obesity risk. In this study, we investigate potential mechanisms by which adult patients' participation in a blood pressure control disease management intervention could produce *spillover* effects on the adolescent family members (adolescents) who live with them. We use guiding hypotheses grounded in existing research literature regarding mechanisms by which parental involvement has been shown to be an effective component of childhood obesity prevention and treatment interventions. Based on the existing literature, this qualitative study was developed with the guiding hypotheses that adult patients (index patients) would identify the following salient mechanisms for producing *spillover*: role modeling of healthy diet and physical activity behaviors, changes to the home food environment, changes in family meal preparation, and improvements in family functioning.

## Methods

### Study Sample/Population

This study was approved by the Johns Hopkins Medical Institutions Institutional Review Board. Adult patients with uncontrolled hypertension who participated in the Achieving Blood Pressure Control Together Study (ACT Study) comparative effectiveness trial were recruited to participate in an in-depth interview following completion of a 12-months follow-up questionnaire (32). The ACT Study tested the relative effectiveness of social and behavioral interventions to improve BP control among an urban, low-income African-American population in Baltimore. The intervention components were tailored and combined using principles of community based participatory research with the goal of improving their effectiveness and sustainability in the specified patient population. All three intervention arms involved blood pressure self-monitoring training plus the use of community health workers. One arm also involved a brief patient and family activation intervention known as “Do My Part.” This intervention was delivered to patients one-on-one in the clinical setting and could have also included an adult family member if he/she was accompanying the index patient to a medical visit but did not involve child or adolescent family members. Another arm also involved an 8-week-long problem-solving intervention delivered to index patients in groups.

Index patients who indicated willingness to be contacted for future research were re-contacted to participate in in-depth interviews that were conducted at their home or in a private conference room at the primary care clinic where index patients received primary care services. Index patients were eligible if they: (1) had completed the 12-months follow-up questionnaire after participating in one of the three arms of the ACT Study and (2) lived with at least one adolescent family member age 12–17 years old who was also willing to be interviewed. Adolescent respondents were eligible if they were 12–17 years old, self-identified as African American, and lived in a household with an index patient participating in an in-depth interview. Index patients and adolescents were recruited, and all interviews were conducted between September and November 2015. Informed consent was obtained by all index patients and primary caregivers of adolescents. Adolescents provided assent prior to study participation as well. Adult index patients were remunerated fifty dollars and adolescent family members forty dollars for their study participation. Interviews were audio recorded and were conducted one-on-one and separately for index patients and adolescent family members.

### Measures

In-depth interview guides were developed for both index patient interviews and adolescent interviews. The interviews were conducted by trained staff. In-depth interviews focused on potential mechanisms by which ACT Study involvement might produce *spillover* effects on diet and PA behaviors among adolescents and whether family-level behavioral and environmental factors could mediate these effects. The major domains discussed included the general experience of ACT Study involvement, descriptions of the relationship between index patients and adolescent family members, role modeling of healthy diet and PA behaviors by index patients, and the influence of index patient participation in the ACT Study on family food environment, family meal preparation, and family functioning. The interviews were intended to explore the a priori hypotheses regarding mechanisms by which the ACT Study interventions were likely to produce spillover effects on adolescent family members' health behaviors.

Table 1 presents some of the questions included in the index patient and adolescent interview guides to provide examples of the types of questions asked and the content covered in these interviews. Of note, interviewers probed as appropriate based on index patients' and adolescents' responses to glean the richest and most complete picture possible of the relationship of index patients' participation in the ACT study and potential spillover effects on their adolescent family members' health behaviors. The goal was to engage index patients and adolescents in a dialogue around the topics outlined in the interview guide. The specific questions asked varied by interview and were responsive to topics discussed by the index patient and adolescent respondents. Interviews were audio-recorded using a digital recording device. They were subsequently transcribed verbatim. Transcripts were cleaned and verified by a study staff member prior to analysis.

**Table 1:** Example questions in ACT study index patient and adolescent interview guides.

**Theme**	**Role modeling**	**Changes to the home food environment**	**Changes in meal preparation**	**Family functioning**
Question	Sometimes we look to others for examples of how to make healthy choices about what we eat or how active we are. Who do you look to for examples about how to live a healthy lifestyle?	What kinds of foods are available in your home? Since you/your family member participated in the ACT Study, how has anything about the food available in your home changed?	What kinds of foods do you eat for meals like breakfast, lunch, and dinner? Since you/your family member participated in the ACT Study, how has anything about mealtime in your home changed?	Have you noticed any changes in the way members of your family get along with one another since you/your family member participated in the ACT Study? If so, what are these changes?

### Data Analysis

Verbatim transcripts from the audio-recorded in-depth interviews and typed field notes from team debriefs after interviews were reviewed prior to analysis. Interview transcripts were coded and then analyzed to identify key themes. Analysis included both deductive hypotheses drawn from the interview guides and inductive findings that emerged from the interviews. A trained member of the study team created code books for use in coding index patient and adolescent interview transcripts with *a priori* codes drawn from the in-depth interview guides and emergent codes based on initial analysis of interview transcripts. The codebook was refined using an iterative process and then applied to all transcripts. Two independent coders trained in qualitative research methods read and coded index patient interview transcripts (KJ and TY). Codes were grouped into categories based on *a priori* and emergent themes. The study team used ATLAS.ti 7 Software to facilitate interview transcript coding and data management (33).

Disagreements in coding were discussed with the study PI (RT). The adjudication process was tracked for all transcripts. The index patient codebook underwent iterative revisions during coding to better capture data relevant to the study's research questions. Analytical coding reports for index patient interviews were generated and organized by theme and discussed between the study PI and the research team. One independent coder read and coded adolescent family member interview transcripts. A second study team member analyzed the coded adolescent transcripts to identify potential areas of convergence or divergence with index patient interviews. Memos were used to document the iterative process of codebook development and emergent themes.

Thematic analysis of the interviews explored the mechanisms through which the parent study may have produced *spillover* effects, including role modeling of healthy behaviors and changes to the home food environment and meal preparation. Analysis also explored the relationship of family functioning with the index patient's disease management and behavior change. Emergent themes were also developed through this process. The adolescent interviews were coded and analyzed to compare perspectives related to *a priori* and emergent themes with the index patients' interviews.

## Results

Twenty-six percent (41 of 159) of index patients enrolled in the ACT Study reported having children in the household ranging in age from 2 to 17 years old (mean = 10.6 years old). The number of children per household ranged from 1 to 5 (mean = 1.7). The mean age of index patients was 54 years old (range 34–75 years old). Sixteen index patients were in the CHW arm, 13 in CHW plus problem solving classes, and 12 in CHW plus Do My Part. Relationships between index patients and child or adolescent family members fell into eight relationship categories: grandmother-granddaughter, grandmother-grandson, grandfather-grandson, mother-daughter, mother-son, stepmother-stepdaughter, father-daughter, and father-son. Among those ACT Study index patients who agreed to be re-contacted for future research, there were 21 who reported living with at least one adolescent family member ages 12–17 years old. Thirteen of these index patients expressed interest in completing in-depth interviews. Ultimately, 11 index patients and 11 adolescents were consented and completed one-on-one in-depth interviews as part of this study.

These 11 ACT index patients consenting to an in-depth interview were similar in age to all ACT index patients living with child or adolescent family members; and the children and adolescents in their household were also of similar age to all children living with ACT index patients. The mean age of ACT Study index patients consenting to the sub-study was 54 years old (range 42–70 years old). Adolescents who participated in this sub-study were between the ages 12–17 years old (mean = 14.3 years old).

Consistent with *a priori* hypotheses, index patients and adolescents described role modeling (Theme 1), changes in the food environment (Theme 2), and changes in meal preparation (Theme 3) as areas where index patient's participation in the ACT Study produced *spillover* influences on adolescent behavior. Counter to a priori hypotheses, however, physical activity and changes to family functioning were not identified by index patient or adolescent interviews as salient mechanisms for *spillover*. Two emergent themes relating to *spillover* were also identified: perceptions of caregiver influence on adolescent family members' health behavior (Theme 4) and adolescent caretaking behaviors (Theme 5). The former (Theme 4) was frequently described in index patient interviews as related to *spillover* mechanisms, and the latter (Theme 5) emerged in the context of discussions regarding adolescents' recognition of index patients' health status, respectively.

### Theme 1: Role Modeling

Role modeling was very salient to index patients' descriptions of how they thought their participation in disease management interventions produced *spillover* on adolescents' health behaviors. Most of the role modeling discussions focused on healthy eating behaviors and not physical activity. Index patients described serving as role models to adolescents but also to other friends and family members in the area of disease management. One index patient and father describes his own dietary changes and how he used his behavior change as motivation to also improve his daughter's diet:

“*And sometimes we get off the wagon, go to McDonald's or something like that, yeah, sometimes. But not regular. She [adolescent family member] loves McDonald's. I'm trying to teach her eventually if you keep going, you're going to have high cholesterol. We try to avoid all of that fast food and if we do go, I get the wrap…I see they got the grilled chicken and stuff like that, and that's about it.” 66 years-old father of daughter*

This disease management role modeling also seems to provide another avenue for educating adolescents about chronic disease. Some index patients describe getting support from their adolescents to engage in disease management related behaviors. These index patients articulate a sense that engaging their adolescents in this process could help them avoid similar health challenges as adults:

“*It's just m[e] and my daughter are the only two with high blood pressure, so he [adolescent] [is] on her about [taking] her meds, and I'm on her about [taking] her meds. And they talk to me, you take your medicine, you know, this that and the third. But we basically, you know, we concerned about each other health issues. So, we stick together with the health thing….”*−*50 years-old grandmother of grandson*

Thus, index patients made connections between being in the ACT study and influencing health behaviors within their families. They identified ways in which they served as a role model to encourage *spillover* of positive health behavior changes they made in response to participation in the disease management interventions that were part of the ACT study. Yet they also saw their condition as a warning of sorts to adolescent family members and hoped that they could encourage adolescents to make healthy choices so as to prevent or delay onset of illnesses, such as hypertension.

### Theme 2: Changes to the Home Food Environment

Changing the home food environment is often discussed by index patients. Strategies for changing the home food environment described by index patients include avoiding certain foods, changing the environment around mealtimes, and making healthier choices when purchasing meals and snacks for consumption at home. One index patient and stepmother describes judiciously avoiding meats that she knows can negatively affect cardiovascular health:

“*… But now, you know, I'm more in tuned to my health. I take my vitamins, you know. I exercise, you know. I watch what I eat. I don't eat no beef. I don't eat no pork. It's strictly like chicken, turkey and seafood, you know…”*−*46 years-old stepmother of stepdaughter*

Another index patient and grandmother specifically describes efforts to change the dynamics in the household around mealtimes and how these have helped her to stick to eating foods that she knows are healthy but that she may find less palatable than the unhealthy foods that adversely affect her blood pressure control:

“*…Everybody ate at a separate time, you know, everybody ate at their own time. And now that I'm retired and I'm home, uh, we were finding less time to eat together too, but then, uh, when I joined the ACT study, we just, we just sat down and ate together you know. We'd just sit down and eat together. Um, and, um, that way, um it's very distracting, as I said, from what you're eating, especially if it doesn't have the taste that you're used to. So, uh, we find it beneficial for both reasons, health reasons and then, uh, you know, just to sit down and have conversation or whatever.”*−*72-years-old grandmother of granddaughter*

Another index patient discussed the way that his increased awareness of health vs. unhealthy foods affects food purchases during shopping trips with his son:

“*He [IFM] goes to the market with me sometimes. Well, the majority of the times, he [adolescent family member] did go to the market with me, because I need him to carry the bags. But he goes. He's getting better because he doesn't grab junk like he used to. He might grab chips and he might grab—he doesn't grab junk like he used to. He'll probably grab some Twizzlers and that's it, and some chips. He grabs granola bars now, and he's drinking a lot of water, so he's better with that. So, I just really—I noticed it but it didn't really dawn on me. But he's getting better with grocery shopping.”*−*44 years-old father of son*

As these examples demonstrate, some index patients discussed removing certain items from the home entirely or limiting purchases of certain foods, and others changed the environment around meal times in an effort to improve dietary behaviors of their whole household, thus facilitating *spillover*. Respondents describe these approaches as directed toward improving their own health but also as a means of creating a home environment that fosters healthier eating behaviors among their adolescents and other family members in the household.

### Theme 3: Changes in Meal Preparation

Given the varied responsibilities of adolescents in the household, the issue of meal preparation was frequently discussed. Index patients describe different approaches to addressing needs for healthier meal preparation techniques for themselves as a means of supporting blood pressure control. Some families adopted a policy of making two separate meals, and others modified meal preparation techniques for the entire household. In several cases, adolescents were actively involved in cooking and meal preparation. As a result, they also learned about and implemented healthier meal preparation methods based on input from index patients:

“*They cut down on the, uh, the fried food. Most of the stuff we grill or bake. So, you know, it calls attention to what they eat too…*.

“*But she, she [adolescent] does a lot of cooking, uh, for me. So, when she cooks, I come down and keep her company. While she cook, she may ask me, you know, so if I put this in there, will it do this, or will it do that? And you know, and I have to tell her, you know, the best, and know how, um, what's right, and what's not right, what's good for me, and what's not. But she, she eats, uh, a lot of the times what I eat too*.”−*72 years-old grandmother of granddaughter*

As this example illustrates, through their involvement in cooking for index patients, adolescents gain insight into healthier cooking techniques, which has the potential of producing *spillover* effects on adolescents' dietary behaviors, particularly when they eat the same foods that they cook for index patients.

### Emergent Themes

#### Theme 4: Varying Perception of Caregiver Influence on Adolescent Family Members

While there were many examples from index patient interviews describing their efforts to produce *spillover* effects of their behavior changes on adolescents, index patients expressed varying levels of confidence about their ability to influence adolescents. Index patients' attitudes about the extent to which their behavior changes influenced adolescents may have affected parental engagement and the likelihood of giving advice. As such, index patients expressed a wide range of opinions about their ability to influence adolescents' health behaviors. Some index patients were intentional about changing adolescents' behaviors and felt they exerted a lot of influence while others were tentative about changing adolescents' behaviors and skeptical of their ability to influence adolescents because they believe they would be ineffective.

In general index patients fell into three categories along a continuum with respect to parental influence: (1) strong influence (i.e., index patients describe intentional efforts to influence adolescents' health behaviors because they felt they were highly influential in adolescents' health behavior decisions), (2) moderate influence (i.e., index patients did not intentionally influence adolescents' health behaviors but did observe changes in adolescents' behaviors that they associated with their own behavior change), and (3) limited influence (i.e., index patients explicitly described that they were ineffectual in influencing adolescents' health behaviors and were unaware if their own health behavior changes had any influence on adolescents). Yet, we found relatively consistent references in adolescent interviews to the fact that adolescents paid attention to what index patients were doing and saying about health behaviors. And, in many cases, we found that the actions and recommendations of index patients influenced adolescents to either make changes, or contemplate health behavior change.

Table 2 presents the index patients' perception of their influence on adolescents' behaviors juxtaposed with exemplar quotes from adolescents, thus representing perspectives from both members of the dyad. These examples are used to demonstrate observed variation in the extent to which index patients perceive that adolescents internalized messages about health behavior change. Because of the contrast between index patient and adolescent perspectives for this emergent theme, Table 2 includes paired quotes from index patient and adolescent interviews.

**Table 2:** Exemplar quotes: parental influence and teen behavior change.

**Parental sense of influence**	**Parent exemplar quote**	**Corresponding adolescent family member quote**
Strong	“I mean, I take my other kids, too, so they'll know also, but I mostly tell him [teen], you go, you get certain things. I also let him know what I'm looking for, he'll read the label, and everything on the box. If it doesn't say what I want, he doesn't bring it back to me, cause he knows that's not what I want” −43-years-old female (mother)	**I:** Who do you look for, or who do you look to for examples on how to be healthy? **F:** My father and my mother …. Like sometimes she'll [index participant] say I need to stop eating a lot of junk food and stuff, and like I can't have junk food every day, and I need to–even though I'm gaining weight, I need to stop doing that.−12-years-old male (son)
Moderate	“… she [adolescent family member] watch what I eat. She know that I don't eat no beef, or no pork…She know I don't do the salt at all. So she, she pays attention. I may not think she paying attention, but she is. She is.”−46-years-old female (stepmother)	Because the way how she [index participant] eat is so healthy, like she eat weird healthy food, like, uh, asparagus and stuff like that. I'm like I wonder how that taste. Like she be, she make sure we eat a lot of healthy food, and make sure we drink a lot of water…”−16-years-old female (stepdaughter)
Limited	“so, in terms of [adolescent family member], he can fry it up. He can fry anything he want to fry. I tried to get him away from frying, but he he's got to fry his food. Got to fry those potatoes, and for chicken nuggets, I try to get him to put them in a pan, stick them in the oven, and heat them up that way, but he won't do it.”—66-years-old male (grandfather)	He [index participant] likes to like enforce us eating healthier foods. Like he always tells me—us to eat healthy and say I should always eat fruit. He thinks I don't eat fruit but I do…. I just—like, he buys a lot of bananas and stuff. Like I like oranges and stuff like that, so like when he always buys bananas—like I'll eat a banana, but like not regularly. But like he'll say I don't eat any fruits or whatever because I don't eat the bananas…”−15-years-old male (grandson)

#### Theme 5: Adolescent Caretaking of the Parent

In addition to describing the ways in which their own attempts at health behavior change influenced adolescents; some index patients described adolescents as being in a caretaking role. Some index patients felt that this caretaking role was another way that disease management programs produced *spillover* effects on adolescents because they were familiar with the health behaviors that were required to manage high blood pressure and prevent adverse health outcomes related to hypertension (Table 3). While not specific to healthy dietary or physical activity behaviors, one index patient described her grandson's knowledge about the importance of medication adherence and blood pressure monitoring and his caretaking role in reminding her of the importance of these:

“*First thing he [adolescent] will ask is did you take your medicine granny? I'm like yeah. I took my medicine. You take your pressure today. Yeah, I took my pressure. Was it normal? Was it high? Oh, it was okay. Then you know, he'll go get the blood pressure cuff. Then he'll turn it on. Then he'll read it. Oh, that was good. It's like such and such points lower than what it was yesterday. Now, you know, this is better than what this reading was all week, you know. He's a good boy and, you know, he's concerned about everybody's health, yeah, very much.”*−*50-years-old grandmother of grandson*

**Table 3:** Exemplar quotes: adolescent caretaking behaviors.

“And when I set her down and explain to her ‘[adolescent family member],' I said, ‘I'm sick.’ ‘No, daddy, you've got to do things. That's what's wrong. If I do everything, you'll just lay down and don't do nothing.’ Like hey, I'm going to make you work. I said, ‘No, you do your part and Daddy going to do his part.”’—66-years-old male (father)
*“[Other child in household] always concerned about my health, always concerned. How you feel? Um, you need some water. Um, um, you take your needle shot (laughter). I'm like yeah [other child in household]. Yes [other child]. Yes [other child]. You know, he, very concerned, and when I was going to the Saturday meetings, he was like you coming back. I'm yeah, I'm coming back, you know. And, ‘cause he was just so worried that I was going to go in the hospital, you know. So it, it really helped. And he see how I eat now, you know. And I be telling him boy, I'm getting ready to, getting ready to put you on that diet with me. Sure is, ‘cause he is, he done out grew all his clothes, new clothes that we just bought him he can't fit at all. I'm like yeah, you, getting ready to do some exercises and all that with me. And he be like okay (laughter). He, that's my boy. That's, he like my right hand man. I take him everywhere.”−46-years-old female (stepmother)*
“That's the main time when I see him [adolescent family member], when he needs something. But as far as that, he really doesn't bother me because he knows my condition, because he knows I'm probably upstairs, with oxygen. And he doesn't really bother because I'm on a lot of medication and stuff. So if he needs something, he comes upstairs, or if he wants something. But as far as that, he'll come to say hi.”—*44-years-old (father)*

Implicit in many descriptions of adolescent caretaking behaviors is this notion that involvement in the ACT study not only made index patients more aware of the need to improve their health behaviors but it provided an avenue by which adolescent family members had a better understanding of the types of behaviors that would help keep their family members healthy and they wanted to be involved in supporting disease management.

### Additional Findings

Two hypothesized *spillover* mechanisms were not corroborated by index patient or adolescent interviews: changes in physical activity, and changes in family functioning due to study participation. Family functioning in particular was not the primary focus areas of most disease management interventions included in the ACT study. Yet, while the issue of family functioning associated with the parental role was discussed explicitly by some, there were also implicit references to family challenges as a source of stress. And some index patients made a clear connection between family stress, blood pressure control, and health behaviors. As one father put it:

“*I'm stubborn, I've got to the point now I hollers a lot at her [adolescent]. Instead of talking to her, I'll holler and—because it seems that when I tell her to do something, she don't even do it. And then before I knew it… I'll be done blowed up and then that's run the pressure up a lot. And then I have to count to 10. And say hey, it's a child you're talking to, count to 10.”*−*66-years-old father of daughter*

This recognition that role stress can affect blood pressure may suggest that there is untapped potential for family-engaged disease management interventions. Such interventions may provide support to index patients and family members to address role stress and family dynamics that produce physiologic and behavioral responses to stress that are counterproductive to achieving blood pressure control and maintaining healthy diet and physical activity behaviors. While interesting and provocative, however, this sentiment was not articulated as clearly as the themes described above.

## Discussion

We found that index patients see themselves as important role models of behavior change for adolescent family members. We also found support from index patients' reported perceptions that their participation in a disease management intervention may produce *spillover* effects on adolescents' dietary behaviors, particularly via role modeling, changes in the home food environment, and meal preparation. These changes were perceived to be associated with increasing knowledge and awareness of healthy eating behaviors among adolescents in addition to improving index patients' own awareness of healthy foods and meal preparation techniques. In contrast, we did not find evidence to suggest significant spillover effects on adolescents' physical activity behaviors. While, there were a few interviews in which physical activity was briefly discussed, in general much more of the discussion focused on dietary behaviors. While it is possible that this difference in spillover for dietary vs. physical activity behaviors reflects a difference in the extent to which these behaviors are susceptible to intergenerational spillover, more likely these differences reflect the fact that the ACT study placed significant emphasis on promoting dietary behavior change as a method for improving blood pressure control (e.g., via intervention messages intended to decrease index patients' consumption of sodium and fried foods).

Because this study relied entirely on data from interviews with index patients and adolescents, we are unable to corroborate index patients' and adolescents' perceptions with objective data related to adolescents' health. Ideally future research would measure adolescents' perceptions of spillover alongside use of objective measures to assess their health behaviors and their objective health status including anthropometric measurements, assessment of weight status, and potentially evaluation of blood pressure and other clinical indicators, such as blood glucose and cholesterol levels.

Beyond these hypothesized mechanisms for adult disease management program participation producing *spillover* effects on the behaviors of adolescent family members, many index patients described an explicit effort to encourage adolescents to adopt healthier lifestyle behaviors. We also found that, despite variation in index patients' perceptions of their influence over adolescents, most adolescents recalled the advice related to diet behaviors that index patients passed onto them after participating in disease management programs. Thus, this study provides evidence to suggest that adolescents are internalizing the new knowledge and behavior changes of their adult family members acquired via disease management program participation. Furthermore, some adolescents employ new skills and disease management techniques to support disease management and minimize negative health outcomes for their adult family members by taking on an active caretaking role, reminding index patients of what they should do to support their own health.

Findings from this research suggest that disease management interventions addressing health behaviors among high-risk populations have untapped potential to produce family-level influences on adolescent health behavior change. This research also suggests a need for more focus on ways to increase family engagement in disease management programs. We argue, in particular, that such family-engaged or family level intervention strategies could increase the influence of disease management interventions over the life course in addressing CVD disparities. It could also be important for reducing childhood obesity and long-term CVD risk among high-risk children and adolescents living with adults family members with chronic disease who may be particularly motivated to prevent intergenerational transfer of CVD risk via health behavior change.

For example, work by Grandes and colleagues suggests that the presence of comorbid conditions associated with obesity can affect adult patients' readiness for behavior change (34). Rhee and colleagues have also found the same regarding parents' receptiveness to addressing obesity in their child (35). Thus, developing family-level disease management programs and increasing family engagement in existing programs targeting high-risk populations may be particularly important as a strategy for engaging adults living with child family members because they are motivated to change their own behavior when they are confident they can also influence health behavior change in other family members, particularly children and adolescents.

This research is exploratory and, while it suggests avenues for further inquiry, these findings are hypothesis generating and should not be viewed as exhaustive in terms of the mechanisms by which *spillover* may operate in the context of disease management programs. Furthermore, given the qualitative methodology of this study, the findings cannot be used to generalize to a broader population of families. Because we did not collect objective measures of health behaviors or clinical indicators, these findings merit further exploration and verification via more rigorous research that links self-reported perceptions of spillover with health behavior data and clinical measures for both adult patients and adolescent family members.

Additionally, there are risks that some responses were influenced by social desirability bias since index patients were educated about healthy behaviors via participation in the ACT study. Similarly, adolescent family members may have been influenced by social desirability bias. There is also a risk that interviewers who conducted in-depth interviews inadvertently gave index patients and adolescents cues regarding the types of responses they were expecting. This could further compromise the validity of the study findings. Future research should involve more in-depth use of qualitative and quantitative methods, potentially over time during implementation of a family-level intervention so as to prospectively evaluate both perceptions of spillover and objective measures of health behavior change and clinical outcomes. Such a study is important for better understanding the timing of adolescents' perceived health behavior changes and the relationship between the changes and the family-level disease management intervention. Ideally such research would also corroborate reports of behavior change using clinical indicators to track changes in weight status and diet composition. Future research should also involve larger patient and family populations and pay special attention to evaluating the factors and circumstances that are associated with both the presence and the absence of spillover effects among some adolescents. In particular, research to better understand the circumstances that prevent spillover effects on adolescents would be important to understand in more detail as understanding conditions that prevent effective spillover effects could inform future intervention development efforts.

Despite these caveats, we believe these findings are intriguing and warrant further, rigorous investigation and that they provide evidence to support the hypothesis that adult disease management interventions may produce *spillover* effects on adolescent family members. Future research should not only rigorously evaluate *spillover* effects of existing disease management programs on child family members, but it should also involve development, testing, and implementation of family-focused disease management programs that explicitly target adult chronic disease management and primordial or primary prevention via health behavior change among child family members. While potential for social desirability bias is important to consider, Our findings were generally consistent with existing literature about parental influence on adolescents. Future interventions could bolster parental confidence in their influence on their adolescent family members and help empower parents to exert role modeling and influence their child's behavior.

## Author Contributions

RT made substantial contributions to all phases of the research including conception or design, acquisition of data, analysis and interpretation of data, and drafting and revising the manuscript. TY made substantial contributions to analysis or interpretation of data for the work and drafting the work or revising it critically for important intellectual content. PE made substantial contributions to the conception or design of the work, the acquisition and interpretation of data for the work and contributing to revising this manuscript for important intellectual content. LB made substantial contributions to the conception or design of the work; the acquisition, analysis, and interpretation of data and also involved in critically revising this manuscript for important intellectual content. LC made substantial contributions to the conception or design of the work; and to the acquisition, analysis, or interpretation of data for the work and also made significant contributions to critically revising this manuscript for important intellectual content.

### Conflict of Interest Statement

The authors declare that the research was conducted in the absence of any commercial or financial relationships that could be construed as a potential conflict of interest.

## References

[1] Ogden C Carroll M Kit B Flegal K. Prevalence of obesity and trends in body mass index among US children and adolescents, 1999–2010. JAMA. (2012) 307:483–90. 10.1001/jama.2012.40 22253364 PMC6362452

[2] Lasserre AM Chiolero A Paccaud F Bovet P. Worldwide trends in childhood obesity. Swiss Med Wkly. (2007) 137:157–8. 17370157 10.4414/smw.2007.11707

[3] Ogden CL Carroll MD Curtin LR Lamb MM Flegal KM. Prevalence of high body mass index in US children and adolescents, 2007–2008. JAMA. (2010) 303:242–9. 10.1001/jama.2009.2012 20071470

[4] Keppel K Pearcy J Heron M Is there progress towards eliminating racial/ethnic disparities in the leading causes of death? Public Health Rep. (2010) 125:689–97. 10.1177/003335491012500511 20873285 PMC2925005

[5] Go A Mozaffarian D Roger V Benjamin EJ Berry JD Borden WB Heart disease and stroke statistics−2013 update: a report from the American Heart Association. Circulation. (2013) 127:e6–245. 10.1161/CIR.0b013e31828124ad 23239837 PMC5408511

[6] Lee JM Pilli S Gebremariam A Keirns CC Davis MM Vijan S. Getting heavier, younger: trajectories of obesity over the life course. Int J Obes (Lond). (2010) 34:614–23. 10.1038/ijo.2009.235 19949415 PMC2926791

[7] Richardson L Hussey J Strutz K. Origins of disparities in cardiovascular disease: birth weight, body mass index, and young adult systolic blood pressure in the National Longitudinal Study of Adolescent Health. Ann Epidemiol. (2011) 21:598–607. 10.1016/j.annepidem.2011.02.012 21497518 PMC3251513

[8] Miech RA Kumanyika SK Stettler N Link BG Phelan JC Chang VW. Trends in the association of poverty with overweight among US adolescents, 1971–2004. JAMA. (2006) 295:2385–93. 10.1001/jama.295.20.2385 16720824

[9] Ali MK Bullard KM Beckles GL Stevens MR Barker L Narayan KM. Household income and cardiovascular disease risks in U.S. children and young adults: analyses from NHANES 1999–2008. Diabetes Care (2011) 34:1998–2004. 10.2337/dc11-0792 21868776 PMC3161277

[10] Rossen L Schoendorf K. Measuring health disparities: trends in racial-ethnic and socioeconomic disparities in obesity among 2- to 18-year old youth in the United States, 2001–2010. Ann Epidemiol. (2012) 22:698–704. 10.1016/j.annepidem.2012.07.005 22884768 PMC4669572

[11] Olds T Maher C Zumin S Péneau S Lioret S Castetbon K. Evidence that the prevalence of childhood overweight is plateauing: data from nine countries. Int J Pediatr Obes. (2011) 6:342–60. 10.3109/17477166.2011.605895 21838570

[12] Robert Wood Johnson Foundation Declining Childhood Obesity Rates–Where Are We Seeing the Most Progress? (2012). Available online at: www.rwjf.org/healthpolicy; http://www.rwjf.org/content/dam/farm/reports/issue_briefs/2012/rwjf401163

[13] U.S. Preventive Services Task Force. The Guide to Clinical Preventive Services 2010–2011 (2010). Available online at: http://www.ahrq.gov/clinic/pocketgd1011/pocketgd1011.pdf

[14] Danaei G Rimm E Oza S Kulkarni S Murray C Ezzati M. The promise of prevention: the effects of four preventable risk factors on national life expectancy and life expectancy disparities by race and county in the United States. PLoS Med. (2010) 7:e1000248. 10.1371/journal.pmed.1000248 20351772 PMC2843596

[15] Ho M Garnett S Baur L Burrows T Stewart L Neve M. Effectiveness of lifestyle interventions in child obesity: systematic review with meta-analysis. Pediatrics (2012) 130:e1647–71. 10.1542/peds.2012-1176 23166346

[16] Hingle M O'Connor T Dave J Baranowski T. Parental involvement in interventions to improve child dietary intake: a systematic review. Prev Med. (2010) 51:103–11. 10.1016/j.ypmed.2010.04.014 20462509 PMC2906688

[17] Foltz JL May AL Belay B Nihiser AJ Dooyema CA Blanck HM. Population-level intervention strategies and examples for obesity prevention in children. Annu Rev Nutr. (2012) 32:391–415. 10.1146/annurev-nutr-071811-150646 22540254 PMC10880737

[18] Grzywacz JG Almeida DM McDonald DA Work-family spillover and daily reports of work and family stress in the Adult Labor Force. Fam Relat. (2002) 51:28–36. 10.1111/j.1741-3729.2002.00028.x

[19] Sturge-Apple ML Davies PT Cicchetti D Cummings EM. The role of mothers' and fathers' adrenocortical reactivity in spillover between interparental conflict and parenting practices. J Fam Psychol. (2009) 23:215–25. 10.1037/a0014198 19364215 PMC2909036

[20] Williams KJ Alliger GM Role stressors, mood spillover, and perceptions of work-family conflict in employed parents. Acad Manage J. (1994) 37:837–68. 10.2307/256602

[21] Laroche H Davis M Forman J Palmisano G Reisinger HS Tannas C. Children's roles in parents' diabetes self-management. Am J Prev Med. (2009) 37(6 Suppl. 1):S251–61. 10.1016/j.amepre.2009.08.003 19896027 PMC2811065

[22] Laroche H Heisler M Forman J Anderson M Davis M. When adults with diabetes attempt to drink less soda: resulting adult-child interactions and household changes. J Natl Med Assoc. (2008) 100:1004–11. 10.1016/S0027-9684(15)31436-X 18807427

[23] Klohe-Lehman D Freeland-Graves J Clarke K Cai G Voruganti VS Milani TJ. Low-income, overweight and obese mothers as agents of change to improve food choices, fat habits, and physical activity in their 1-to-3-year-old children. J Am Coll Nutr. (2007) 26:196–208. 10.1080/07315724.2007.10719602 17634164

[24] Lubans D Morgan P Collins C Okely A Burrows T Callister R Mediators of weight loss in the “Healthy Dads, Healthy Kids” pilot study for overweight fathers. Int J Behav Nutr Phys Act. (2012) 18:45 10.1186/1479-5868-9-45 PMC3418215 22512861

[25] Sato A Jelalian E Hart C Lloyd-Richardson EE Mehlenbeck RS Neill M. Associations between parent behavior and adolescent weight control. J Pediatr Psychol. (2011) 36:451–60. 10.1093/jpepsy/jsq105 21112925 PMC3079126

[26] Ma J Flanders W Ward E Jemal A Body mass index in young adulthood and premature death: analyses of the U.S. National Health Interview Survey linked mortality files. Am J Epidemiol. (2011) 174:934–44. 10.1093/aje/kwr169 21873602

[27] Arcan C Neumark-Sztainer D Hannan P van den Berg P Story M Larson N. Parental eating behaviours, home food environment and adolescent intakes of fruits, vegetables, and dairy foods: longitudinal findings from Project EAT. Public Health Nutr. (2007) 10:1257–65. 10.1017/S1368980007687151 17391551

[28] Hanson N Neumark-Sztainer D Eisenberg M Story M Wall M. Associations between parental report of the home food environment and adolescent intakes of fruits, vegetables and dairy foods. Public Health Nutr. (2005) 8:77–85. 10.1079/PHN2004661 15705248

[29] Golan M Parents as agents of change in childhood obesity–from research to practice 65. Int J Pediatr Obes. (2006) 1:66–76. 10.1080/17477160600644272 17907317

[30] Laroche H Davis M Forman J Palmisano G Heisler M. What about the children? The experience of families involved in an adult-focused diabetes intervention. Public Health Nutr. (2007) 11:427–36. 10.1017/S1368980007000791 17686202

[31] Barr-Anderson D Adams-Wynn A DiSantis K Kumanyika S. Family-focused physical activity, diet and obesity interventions in African-American girls: a systematic review. Obes Rev. (2013) 14:29–51. 10.1111/j.1467-789X.2012.01043.x 23057473 PMC3524349

[32] Ephraim PL Hill-Briggs F Roter DL Bone LR Wolff JL Lewis-Boyer L. Improving urban African Americans' blood pressure control through multi-level interventions in the achieving blood pressure control together (ACT) study: a randomized clinical trial. Contemp Clin Trials (2014) 38:370–82. 10.1016/j.cct.2014.06.009 24956323 PMC4169070

[33] Friese S ATLAS.ti 7: User Guide and Reference. Berlin: Atlas.ti Scientific Software Development GmbH (2013).

[34] Grandes G Sanchez A Torcal J Sánchez-Pinilla RO Lizarraga K Serra J. Targeting physical activity promotion in general practice: characteristics of inactive patients and willingness to change. BMC Public Health (2008) 8:172. 10.1186/1471-2458-8-172 18498623 PMC2412873

[35] Rhee K De Lago C Arscott-Mills T Mehta S Davis R. Factors associated with parental readiness to make changes for overweight children. Pediatrics (2005) 116:e94–101. 10.1542/peds.2004-2479 15995022

